# The parenting attitudes and the stress of mothers predict the asthmatic severity of their children: a prospective study

**DOI:** 10.1186/1751-0759-4-12

**Published:** 2010-10-07

**Authors:** Jun Nagano, Chikage Kakuta, Chikako Motomura, Hiroshi Odajima, Nobuyuki Sudo, Sankei Nishima, Chiharu Kubo

**Affiliations:** 1 Institute of Health Science, 6-1 Kasuga Park, Kasuga, Fukuoka, 816-8580 Japan; 2 Department of Psychosomatic Medicine, Kyushu University Graduate School of Medical Sciences, Fukuoka, Japan; 3 Department of Pediatrics, Fukuoka National Hospital, Fukuoka, Japan

## Abstract

**Objective:**

To examine relationships between a mother's stress-related conditions and parenting attitudes and their children's asthmatic status.

**Methods:**

274 mothers of an asthmatic child 2 to 12 years old completed a questionnaire including questions about their chronic stress/coping behaviors (the "Stress Inventory"), parenting attitudes (the "*Ta-ken *Diagnostic Test for Parent-Child Relationship, Parent Form"), and their children's disease status. One year later, a follow-up questionnaire was mailed to the mothers that included questions on the child's disease status.

**Results:**

223 mothers (81%) responded to the follow-up survey. After controlling for non-psychosocial factors including disease severity at baseline, multiple linear regression analysis followed by multiple logistic regression analysis found chronic irritation/anger and emotional suppression to be aggravating factors for children aged < 7 years; for children aged 7 and over, the mothers' egocentric behavior was a mitigating factor while interference was an aggravating factor.

**Conclusions:**

Different types of parental stress/coping behaviors and parenting styles may differently predict their children's asthmatic status, and such associations may change as children grow.

## Background

Although available data to date are limited, notable associations between children's depression and asthma-related deaths [1] and children's chronic stress and asthma attacks have been reported [2]. Clinicians know that there are children, whose symptoms are improved by a "parentectomy", i.e., putting an asthmatic child in a hospitalized setting to separate them from home [3,4]. More recent research shows that the asthmatic morbidity of children may be affected by family functioning [5], their parent's mental health including depression [6-9], or certain types of parenting styles [10,11]. Taken together, parents may, when they are in a stressful situation or when they have certain parenting attitudes, not only reduce support for their children [9], but also become a stressor themselves, which affects the disease status of their child's asthma.

Analyzing videotaped mother-child talk sessions, Hermanns et al found that mothers whose children had more frequent asthma attacks tended to show more critical attitudes to their child [10], although such an association was not found by tape-recorded father-child talk sessions carried out by Schobinger et al [12]. Shibuya et al found that mothers whose parenting styles were of the "passive rejection", "active rejection", or "doting" type tended to have asthmatic children who had more severe airway contraction [11]. They also found fathers with parenting styles of the "sacrifice" or "doting" type to be associated with their child's more severe airway contraction, but such associations were weaker for fathers than for mothers. These findings, however, were all based on retrospective studies, so determining the direction of the association is rather difficult. The association between parental stress and an asthmatic child's morbidity or severity has been reported in retrospective studies [6,7], and also from a few prospective studies [8,9]. Weil et al reported that the primary caretaker's mental health problems predicted the number of hospitalizations during the subsequent nine months for asthmatic children aged 4-9 years old [8]. Bartlett et al found that children whose mother had scored high on a depression scale developed more asthmatic symptoms and visited ED more frequently in the subsequent six months, as compared to those whose mother had not been depressive [9]. Further studies, however, are needed to clarify what specific kinds of stressful parental conditions or parental stress-related properties affect the status of their children's asthma [13].

Other issues to be clarified include the mechanisms that link certain parenting styles and parental stress with a child's disease status. Adherence, i.e., the extent to which children and their caregivers continue health-related behaviors, is thought to be among them, because it has been shown to be affected by parental critical attitudes [14], familial dysfunction [15], or parental mental health [16]. The nature or strength of parent-child relationships changes as children grow, and this could alter the effects of maternal psychosocial properties over the asthmatic status of their children. However, to date little has been reported on this issue.

The primary purpose of this study was to test in a prospective observational setting if "unfavorable" parenting styles have the potential to affect a child's asthmatic morbidity, and to identify the specific stressful conditions of a mother that may affect her child's disease status, considering a variety of possible confounding factors including adherence and other physical, dispositional, clinical, and environmental factors. We also addressed if the effect of a mother's parenting style and stress on her child's asthma is different according to the child's age.

## Methods

### Subjects and data collection

A consecutive series of eligible bronchial asthma patients and their mothers who visited the out-patient clinic of the Pediatrics Department of the National Fukuoka Hospital between February 1 and August 31, 2001 were invited to participate in the study. Criteria for enrollment included: (i) patient age 2 to 12 years; (ii) duration of bronchial asthma one year or longer; (iii) the necessity of repeat visits to the clinic (or hospitalization) over the past year; and (iv) medications necessary on a regular basis for the past year. The mothers of the patients who met the criteria were introduced by the pediatrician in charge to one of the authors. The author explained the study purpose and methods in person, gave instructions for completing the questionnaire, and invited them to participate in the study. The mothers who agreed were then handed the baseline questionnaire and a self-addressed stamped envelope, along with a book coupon of 1000 yen as a token of gratitude. Some of the participating mothers completed the questionnaire and returned it immediately, and the others completed it at home and mailed it back. A reminder call was made when there was no return mail within two weeks. All the participating mothers had learned the basic knowledge about bronchial asthma, including the etiology, mechanisms, diagnosis, treatment, and management, typically through a one-day seminar at the hospital. One year after the baseline survey, i.e., between February 1 and August 31, 2002, a follow-up mail that included the follow-up questionnaire, a self-addressed stamped envelope, and a 1,000 yen book coupon was sent to the mothers who had returned the baseline questionnaire. Data from medical records was also used to obtain clinical information including serum immunoglobulin E (Ig E) level, measured within two years before the baseline survey. The study protocol was approved by the ethics committee of the Institute of Health Science, Kyushu University.

### Questionnaires

The baseline questionnaire included questions about the mother's age, patient's date of birth, mother's occupation, mother's education, cohabiters, age at diagnosis, factors related to deteriorations, comorbid allergic conditions, family history of allergic conditions, intensity and frequency of attacks in the past year, cohabitation with smokers, adherence, medications, mother's parenting style (see below), and mother's stress or coping behaviors (see below). Factors related to deteriorations were assessed by picking relevant items from a list that consisted of five categories with 4-7 items in each, i.e., an infection category with cold, tonsillitis, etc.; an inhalation category with dust, pollen, etc.; a physical category with exercise, fatigue, etc.; a mental category with anxiety, grief, etc.; and a meteorological category with coldness, heat, etc. The list for comorbid allergic conditions included items for atopic dermatitis, allergic rhinitis, food allergy, drug allergy, urticaria, and others. The intensity and frequency of attacks in the past year was assessed using an intensity × frequency matrix, in which the mother was guided to indicate 1-3 combinations (cells) that met the status of her child's attacks (see Additional file 1). This table was designed based on the Japanese Pediatric Guidelines for the Treatment and Management of Asthma (JPGL) 2000 [17], which was developed by the Japanese Society of Pediatric Allergy and Clinical Immunology (JSPACI). Adherence was assessed using a set of four questions: (i) Does your child take or inhale medicines as prescribed by his/her doctor? (ii) Do you take care in order to reduce allergens, such as cleaning rooms and bedclothes? (iii) Does your child visit the clinic regularly as recommended by the doctor? (iv) When an attack happens does your child take or inhale medicines immediately? The answers were given on a 7-point rating scale, 1 to 7, where 1 and 7 respectively correspond to "no" and "yes". The adherence score was the sum of the item scores; Cronbach's coefficient alpha was 0.72 in the present sample. Questions on medications asked about the frequency of use in the past week for each inhaled corticosteroid, oral and inhaled β2-stimmulants, oral theophylline, and oral and inhaled anti-allergic agents.

The follow-up questionnaire included questions about the intensity and frequency of attacks in the past year, adherence, and medications, that were the same as those in the baseline questionnaire.

### Assessment of mother's parenting style

The mother's parenting style was assessed using the "Parent Form" of the "*Ta-ken *Diagnostic Test for Parent-Child Relationship" (T-PCRp) [18]. T-PCRp is a self-administered questionnaire that was developed, based on Symonds' theory of parent-child relationship [19], to assess "unfavorable" parenting attitudes in terms of three axes; "rejection--acceptance", "dominance--obedience", and "inconsistency and disagreement". Additional file 2 shows the construction of this instrument, which consists of five parenting attitudes and two scales corresponding to each of the attitudes. A brief description of key characteristics of the scales is also noted in the table. Each scale consists of 10 questions, and the answers are given on a 3-point rating scale, such as "no", "sometimes" and "always" or "no", "fairly much" and "very much". To each answer, 1, 2 or 3 points were assigned, so that a higher score indicates a stronger tendency. Reliability based on the split-half method was high (r = .90) [18]. Because all 10 scales represent *unfavorable *attitudes toward parenting, we hypothesized that a strong tendency toward any of the attitudes could negatively affect the status of a child's asthmatic symptoms (as indicated by the symbols "•" or "••" in the table of Additional file 2). Special emphasis, however, was placed on three scales, "passive rejection", "active rejection", and "doting", on which mothers of children with strong airway contraction reportedly score high (indicated by "••" in the table) [11].

### Assessment of the mother's stress and coping behaviors

The mother's stress and coping behaviors were assessed using the Stress Inventory (SI). SI is a self-administered questionnaire that was developed, based on Grossarth-Maticek's theory of disease-prone stress/personality [20], to assess response styles to stressors principally related to an interpersonal relationship or to chronic stress posed by the response style [21]. The detailed developmental process and psychometric properties [22,23], brief explanations [24], an English translation of items [25], and some empirical results suggesting its external validity [24,25] are described elsewhere. Additional file 3 presents the 12 SI scales and brief descriptions. Each scale typically consists of four questions, and the answers receive a 1 to 6 rating, where 1 and 6 respectively correspond to "yes" and "no" or to "almost always" and "rarely". Cronbach alphas [26] and test-rest reliability coefficients ranged from 0.60 to 0.90 and from 0.66 to 0.82, respectively [23]. SI was developed principally to assess stress-coping styles or chronic stress that were thought to be risk factors for cancer and cardiovascular disease, and its measured constructs are based on the subject's individual experiences. However, because the stresses that this instrument focuses on are mainly those posed by the pattern of the interpersonal relationship, we thought that some of the patterns might affect those around her through her language and behaviors, especially her children, who are close others. Specifically (as indicated by "••" in the table of Additional file 3), we hypothesized that the following maternal conditions would lead to lowered functioning of care and/or language and behavior that can be stressors to a child, and in turn to negative effects on the child's asthma: (i) a mother's chronic irritation and anger as represented by the SI scales "object dependence of anger" and "annoying barrier", (ii) mother's egocentric and egoistic behaviors as represented by "egoism", and (iii) mother's ambivalent and unstable behaviors, such as anger and depression in interpersonal connections, as represented by "object dependence of ambivalence". We also hypothesized that the following conditions ("•" in the table) would also be predictive of a child's poorer disease status, though more weakly than the above three, because these were thought to be related to lowered functioning of care, but not to stressors to the child: (iv) decreased sense of control over stressful situations ("low sense of control"), (v) chronic sense of hopelessness and depression ("object dependence of loss"), (vi) chronic feelings of unfulfilled needs for acceptance by others ("unfulfilled needs for acceptance"), (vii) altruistic tendency accompanied by stress ("altruism"), and (viii) an extreme tendency to react rationally to stressful interpersonal situations ("rationalizing conflicts/frustrations").

### Analysis

The designated cells in the intensity × frequency matrix in the baseline and follow-up questionnaires were related to the disease severity according to the JPGL 2000 classification, as demonstrated in Additional file 4. When more than one severity was indicated, the highest was adopted. Patients with a condition better than Mild disease were classified as in Remission. Thus, the severity was determined for one of the four categories, Remission, Mild, Moderate, and Severe. A composite variable for anti-asthmatic agents was created according to the JPGL 2000 scoring system, where the medication score was calculated as the sum of points that are given to agents, e.g., 10 points per 5 mg of oral predonisolone, 10 points per 500 μg of inhaled betamethazone, 2 points per 1 oral dose of long-acting theophylline, 1 point per 1 inhaled dose of cromoglycate, etc. An ordinal variable was created for the number of comorbid allergic conditions, where the values were the counts of designated items taken from among atopic dermatitis, allergic rhinitis, and others. An ordinal variable was also created for the number of adult cohabiters, where the values were 1 = mother only, 2 = mother plus 1, and 3 = mother plus 2+.

Statistical analysis was performed in five steps as follows. First, a series of univariate regression analyses was used to identity baseline non-psychosocial factors that were significantly (P <.05) associated with the disease severity at 1y follow-up. Thus, the outcome variable was severity at 1y follow-up (Remission = 1, Mild = 2, Moderate = 3, Severe = 4), and the explanatory variables considered were gender, age at baseline (in years), age at diagnosis (in years), duration (in years), severity at baseline (1, 2, 3, 4), the medication score (continuous), number of comorbid allergic conditions (0, 1, 2, 3), parental history of bronchial asthma (present/absent), number of factors reported to cause deterioration, serum Ig E (logarithm of measurement value), mother's age (in years), mother's education (in years), mother's occupation (housewife/working mother), number of adult cohabiters (1, 2, 3), and living with smokers (present/absent). The above identified variables were then put into a single multiple regression model, in order to identify non-psychosocial covariates that were to be considered for the subsequent analyses of psychosocial factors. Second, a series of univariate regression analyses was performed for psychosocial factors, i.e., mother's parenting styles and mother's stress/coping behaviors. Thus, for each model the outcome variable was the severity at 1y follow-up and the explanatory variable was one of the scale scores of T-PCRp and SI. A series of multiple regression models was then used to adjust for the above identified non-psychosocial covariates. Third, in order to examine a possible confounding effect of adherence on the association between the mother's psychosocial factors and the child's disease severity, both adherence at baseline and adherence at 1y follow-up were added to the series of multiple regression models, including a psychosocial variable and the non-psychosocial factors. Fourth, possible differences in the effects of the mothers' parenting style and stress over their children's asthma according to the child's age were examined by repeating the above analysis, stratified by age categories of < 7 years and 7 and over years (before or after school age). Finally, associations found to be significant in the above stratified analysis were further examined in terms of odds ratio (OR). ORs of moderate/severe disease vs. remission/mild disease at follow-up for high and intermediate categories relative to low category, as classified by tertiles of scale scores, were estimated using multiple logistic regression adjusted for non-psychosocial covariates. All P-values were two-sided, and considered significant at P < .05. All analyses were done using the SAS version 9.3 (SAS Institute Inc., Cary, NC).

## Results

Of the 279 mothers who had orally agreed to participate in the study, 274 (98%) returned a completed questionnaire and a written consent form. One year later, 223 (81%) of the 274 mothers responded to the follow-up mail survey and returned a completed questionnaire; the data from which was used for the present analysis. Table 1 shows the demographic, clinical, and environmental characteristics of the asthmatic children studied. The median (inter-quartile range, or IQR) of the patients' age was 6 (4-8) years, and male patients accounted for 67%. Asthma had developed by the age of 4 in the majority (78%), the median (IQR) for duration was 4 (2-6) years, and the moderate or more severe disease classification by JPGL 2000 accounted for 46%. The percentage of regular medication users ranged from 17% for corticosteroids to 74% for theophylline. Comorbid atopic dermatitis and allergic rhinitis were present in 45% and 58% of the patients, respectively, and 31% were a child of parents with a history of asthma. The median (IQR) of the number of factors reported to cause deterioration was 5 (3-8), and the median (IQR) of serum Ig E was 499 (192-1253) IU/ml. The average age of the mothers was 36 years, the median mothers' education was 14 years, which corresponds to junior-college graduation, and two thirds were housewives. The majority (80%) of the patients lived with two adults, typically mother and father, and more than half lived with a smoker.

**Table 1:** Baseline characteristics (N = 223)

Characteristics	N	(%)*
Demographic factors		
Patient age at baseline		
2-3 y	32	(14.4)
4-5 y	56	(25.1)
6-7 y	57	(25.6)
8-9 y	50	(22.4)
10-12 y	28	(12.6)
Patient sex		
Boys	150	(67.3)
Girls	73	(32.7)
		
Clinical background		
Patient age at diagnosis		
0-<2 y	91	(40.8)
2-<4 y	82	(36.8)
4-<6 y	22	(9.9)
6-<8 y	18	(8.1)
8+ y	10	(4.5)
Duration		
1-<2 y	50	(22.4)
2-<4 y	60	(26.9)
4-<6 y	53	(23.8)
6-<8 y	32	(14.4)
8+ y	28	(12.6)
The JPGL 2000 severity classification:		
Remission	13	(5.8)
Mild disease	107	(48.0)
Moderate disease	84	(37.7)
Severe disease	19	(8.5)
Medications:		
Corticosteroids regular use	38	(17.0)
β2-stimulants regular use	100	(44.8)
Theophylline regular use	166	(74.4)
DSCG inhalation regular use	101	(45.3)
Oral anti-allergics regular use	45	(20.2)
Comorbid allergic conditions:		
Atopic dermatitis	100	(44.8)
Allergic rhinitis	130	(58.3)
Other allergic conditions	103	(46.2)
Parent history of bronchial asthma	70	(31.4)
No. of factors reported to cause deterioration	5	(3-8)^†^
Serum Ig E (IU/ml)	499	(192-1253)^†^
		
Environmental background		
Mother's age (y)	36.2	(5.2)^‡^
Mother's education (y)	14	(9-18)^§^
Mother's job:		
Full-time worker	25	(11.2)
Part-time worker	44	(19.7)
Housewife	147	(65.9)
Others	7	(3.1)
Number of adult cohabiters:		
1 (mother only)	5	(2.2)
2 (mother plus 1)	179	(80.3)
3+ (mother plus 2+)	39	(17.5)
Living with smokers	130	(58.6)

*Figures are in N (%) unless otherwise indicated: ^†^median (IQR), ^‡^mean (standard deviation), ^§^median (range).

Univariate regression analyses found the following non-psychosocial factors to be significantly associated with more severe disease at 1y follow-up (Table 2): Younger patient age at diagnosis, more factors reported to cause deteriorations, more comorbid allergic conditions, higher JSPACI medication scores, higher serum Ig E level, more severe disease at baseline, and working mother compared to housewife. The multiple regression model including all these variables identified only Ig E and disease severity at baseline as the significant (P <.05) factors to be retained in the subsequent analyses of psychosocial factors.

**Table 2:** The association between non-psychosocial factors of asthmatic children and their disease severity in the subsequent year

Factor	Univariate regression		Multiple regression	
		
	β	P	β	P
Patient age at baseline	-.182	.007	-.111	.07
No. of factors reported to cause deterioration	.186	.005	.022	.73
No. of comorbid allergic conditions	.171	.010	.117	.07
JSPACI medication score	.229	<.001	.108	.09
Serum Ig E level	.321	<.001	.181	.039
Disease severity at baseline	.420	<.001	.353	<.001
Working mother vs. housewife	.159	.018	.090	.13

β: standardized partial correlation coefficient. JSPACI: Japanese Society of Pediatric Allergy and Clinical Immunology.

Table 3 shows the association between a mothers' parenting style assessed at baseline and her child's disease severity as evaluated in the 1 y follow-up survey. Univariate analysis identified each of the higher scores of active rejection and interference to be significantly associated with more severe disease at the follow-up. However, when the above retained non-psychosocial factors were adjusted for (Multiple regression 1 in Table 3), only interference was significantly associated with severity at the follow-up. These results did not change after additional adjustment for adherence at baseline and adherence at the follow-up (Multiple regression 2). We then repeated these analyses stratified by the age category (< 7 years, N = 121; 7 years and over, N = 102). Table 4 shows the results of the stratified analyses using models including the significant non-psychosocial covariates (corresponding to Multiple regression 1 in Table 3). For children of age < 7 years, none of the mother's parenting styles was significantly associated with her child's disease status at follow-up, whereas for children of age 7 years and over, stronger tendencies of interference and inter-parental inconsistency were associated with a child's poorer prognosis. The adjustment for adherence did not change either of these associations (data not shown).

**Table 3:** The association between a mother's parenting style and her child's disease severity in the subsequent year

The T-PCRp scale	Univariate regression		Multiple regression 1*		Multiple regression 2^†^	
			
	β	P	β	P	β	P
Passive rejection	.103	.13	.083	.17	.096	.13
Active rejection	.142	.034	.080	.19	.086	.17
Strict control	.033	.63	.010	.87	.014	.82
Overexpectation	.062	.36	.053	.38	.057	.36
Interference	.132	.049	.123	.041	.129	.035
Overconcern	.080	.23	.078	.20	.077	.21
Sacrifice	-.031	.65	-.008	.89	-.009	.88
Doting	-.057	.39	-.046	.45	-.046	.45
Contradiction	.058	.39	.044	.47	.045	.46
Inter-parental inconsistency	.120	.08	.079	.19	.087	.16

T-PCRp: *Ta-ken *Diagnostic Test for Parent-Child Relationship, Parent Form. β: standardized partial correlation coefficient. *Adjusted for serum Ig E at baseline and severity at baseline. ^†^Adjusted for covariates in Multiple regression 1 plus adherence at baseline and adherence at 1 year follow-up.

**Table 4:** The association between a mother's parenting style and her child's disease severity in the subsequent year, stratified by age

The T-PCRp scale	Age < 7 years* (N = 121)		Age 7+ years* (N = 102)	
		
	β	P	β	P
Passive rejection	.100	.23	.083	.36
Active rejection	.112	.20	.077	.39
Strict control	-.065	.44	.021	.81
Overexpectation	.148	.08	.027	.76
Interference	.023	.78	.251	.005
Overconcern	-.030	.72	.169	.06
Sacrifice	.048	.57	-.135	.13
Doting	-.084	.31	-.035	.70
Contradiction	-.006	.94	.069	.45
Inter-parental inconsistency	.007	.94	.190	.030

T-PCRp: *Ta-ken *Diagnostic Test for Parent-Child Relationship, Parent Form. β: standardized partial correlation coefficient. *Adjusted for serum Ig E at baseline and severity at baseline.

Table 5 shows the association between a mothers' stress at baseline and her child's disease severity at follow-up. Univariate analysis identified each of the higher scores of object dependence of anger, annoying barrier, unfulfilled needs for acceptance, and altruism to be significantly associated with a more severe disease at follow-up. When the above retained non-psychosocial covariates were controlled for (Multiple regression 1 in Table 5), the associations of these four SI scales remained significant. The multivariate regression model also revealed an *inverse *association between a mothers' egoism and her child's disease severity. Further adjustment for the adherence factors did not change the associations of these five SI scales (Multiple regression 2). Stratified analysis showed a remarkable contrast between the age categories (Table 6). The above found four risk factors plus disclosure of negative experiences were associated with poorer disease status at follow-up for children aged < 7 years. For children aged 7 years and over, however, while no relation was found for these factors, egoism was *inversely *associated with the severity at follow-up. These findings did not change after controlling for adherence (data not shown).

**Table 5:** The association between a mother's stress/coping behaviors and her child's disease severity in the subsequent year

The SI scale	Univariate regression		Multiple regression 1*		Multiple regression 2^†^	
			
	β	P	β	P	β	P
Low sense of control	.018	.79	.069	.26	.068	.27
Object dependence of loss	.056	.41	.055	.36	.054	.37
Object dependence of happiness	-.012	.85	.007	.91	.004	.95
Object dependence of anger	.176	.008	.201	<.001	.210	<.001
Annoying barrier	.200	.003	.188	.002	.192	.002
Object dependence of ambivalence	.052	.44	.034	.58	.035	.57
Disclosure of negative experiences	-.107	.11	-.096	.11	-.096	.11
Unfulfilled needs for acceptance	.183	.006	.142	.019	.144	.018
Altruism	.173	.010	.190	.002	.194	.001
Egoism	-.081	.23	-.125	.039	-.129	.040
Rationalizing conflicts/frustrations	.067	.32	.039	.52	.039	.53
Lack of emotional experiences	-.022	.74	-.061	.31	-.060	.33

SI: Stress Inventory. β: standardized partial correlation coefficient. *Adjusted for serum Ig E at baseline and severity at baseline. ^†^Adjusted for covariates in Multiple regression 1 plus adherence at baseline and adherence at 1 year follow-up.

**Table 6:** The association between a mother's stress/coping behaviors and her child's disease severity in the subsequent year, stratified by age

The SI scale	Age < 7 years* (N = 121)		Age 7+ years* (N = 102)	
		
	β	P	β	P
Low sense of control	.164	.05	-.039	.66
Object dependence of loss	.062	.46	.042	.64
Object dependence of happiness	.130	.12	-.136	.13
Object dependence of anger	.253	.002	.137	.13
Annoying barrier	.321	<.001	.027	.77
Object dependence of ambivalence	.140	.10	-.101	.26
Disclosure of negative experiences	-.174	.034	-.046	.61
Unfulfilled needs for acceptance	.225	.006	.043	.63
Altruism	.270	<.001	.079	.38
Egoism	-.088	.30	-.198	.027
Rationalizing conflicts/frustrations	.070	.40	-.009	.92
Lack of emotional experiences	-.027	.74	-.101	.27

SI: Stress Inventory. β: standardized partial correlation coefficient. *Adjusted for serum Ig E at baseline and severity at baseline.

Table 7 shows the above associations that were found to be significant in the stratified analyses in terms of OR estimates, adjusted for non-psychosocial covariates. For children aged < 7 years, the ORs of moderate/severe disease at follow-up for the intermediate and high scores of object dependence of anger (SI) were 1.8 and 5.5, respectively, compared to the low score. The ORs for the high score of the risk factors ranged from 3.8 (unfulfilled needs for acceptance) to 13.6 (annoying barrier), with a lower confidence bound > 1.0 and a significant dose response relationship. However, the confidence interval of the OR for the high score of disclosure of negative experiences included unity, and the trend was not clear. For children aged 7 years and over, the ORs for the high scores of interference and egoism were 4.3 and 0.21, respectively, compared with the low scores, and these associations were statistically significant. The association of inter-parental inconsistency was, however, less clear and insignificant in terms of OR.

**Table 7:** The ORs for moderate/severe disease vs. remission/mild disease at follow-up for the baseline levels of selected mother's parenting styles and mother's stress/coping behaviors

Questionnaire:	Scale score			P trend
		
Scale	Low*	Intermediate	High	
*Age <7 years*				
SI:				
Object dependence of anger	1.0	1.8 (0.64-4.9) ^†^	5.5 (1.8-16.8)	.003
Annoying barrier	1.0	2.7 (0.79-9.2)	13.6 (3.6-50.8)	<.001
Disclosure of negative experiences	1.0	0.29 (0.10-0.87)	0.47 (0.17-1.28)	.17
Unfulfilled needs for acceptance	1.0	1.4 (0.52-3.9)	3.8 (1.3-11.0)	.014
Altruism	1.0	3.3 (1.1-10.3)	6.5 (1.9-22.2)	.003
*Age 7+ years*				
T-PCRp:				
Interference	1.0	0.96 (0.32-2.8)	4.3 (1.3-13.6)	.019
Inter-parental inconsistency	1.0	0.90 (0.30-2.7)	1.9 (0.67-5.6)	.23
SI:				
Egoism	1.0	0.33 (0.10-1.1)	0.21 (0.06-0.66)	.009

OR: odds ratio. SI: Stress Inventory. T-PCRp: Ta-ken Diagnostic Test for Parent-Child Relationship, Parent Form. *Reference category. ^†^OR (95% confidence interval), using multiple logistic regression model adjusted for serum Ig E at baseline and severity at baseline.

## Discussion

The present study partly supported the notion that some "unfavorable" parenting styles have the potential to predict a poorer disease status of a child's asthma and identified several specific types of a mothers' chronic stress and specific patterns in terms of stress coping behavior that may predict a poorer or better status of her child's asthma. In addition, it showed that these associations between a mother's psychosocial properties and her child's asthma are considerably different according to the child's age.

Previous studies suggested that a child's more severe asthmatic status was associated with its mother's more critical attitudes to the child [10], and to higher parenting attitudes of passive rejection, active rejection, or doting [11]. However, these studies were based on retrospective data, and interpretation of causality is rather difficult. The present study showed that for children aged < 7 years, a mothers' high active rejection (standardized partial correlation coefficient β = .22, P = .008) or interference (β = .17, P = .034) score was correlated with more severe disease *at baseline*, but not at follow-up (Table 4). However, for the children aged 7 years and over interference and inter-parental inconsistency, which were not correlated with the baseline severity (data not shown), were positively associated with the severity *at follow-up*, suggesting the importance of prospective observation in examining such relationships.

The present study identified some factors related to the chronic stress and stress-coping behaviors of mothers that are associated with a child's asthmatic status in the subsequent year. Reportedly, the disease severity of an asthmatic child may decrease the asthma-associated quality of life of parents [27], and a child's hospitalization in the past 12 months may increase a care-taker's depression or anxiety [28]. In the present study, however, none of the maternal stress factors significantly related to a child's asthma at follow-up was materially associated with the severity at baseline (data not shown), except for a positive correlation with unfulfilled needs for acceptance for children aged < 7 years (β = .21, P = .011). Moreover, the associations between a mother's stress at baseline and her child's disease severity at follow-up remained after controlling for the severity at baseline. It is likely that the mothers' stress at baseline affected her child's disease status in the subsequent year. Wright et al reported, in a prospective study that followed asthmatic infants aged 2-14 months, that while parental stress predicted a child's wheezing, a child's wheezing did not affect parental stress [29].

For the children aged < 7 years of the present study, a mother's stress/coping behaviors, especially chronic irritation and anger represented by the SI object dependence of anger and annoying barrier scales, and emotion-suppressive coping behavior represented by the SI unfulfilled needs for acceptance and altruism scales, were predictive of a more severe disease in the subsequent year. Because adjustment for adherence did not change these associations, it is unlikely that the association between a mother's stress and her child's disease was mediated by the mother's impaired care-taking functions. A mothers' stress (or wellbeing) may be verbally or non-verbally conveyed to her child, and affect the child's asthmatic status via a psycho-physiological pathway, such as by immunoreactivity to allergens or a vulnerability to airway infections. Research shows that while sadness induces cholinergic airway contraction, happiness causes airway relaxation [30] and that cortisol response to psychosocial stress is blunted in asthmatic children [31].

For the children aged 7 years and over, a child's asthma was not associated with the mother's stress relevant to chronic irritation or emotional suppression, suggesting that as children grow their allergic conditions become less affected by communication with their mother, even those who are under chronic stress. However, a higher egoism score was *inversely *related to the severity at follow-up, which was a rather unexpected finding. Among the parenting styles, a strong tendency toward maternal interference was related to a poor prognosis for asthmatic children. These associations were not altered by adjustment for adherence. For older children, a mother's egocentric, self-defensive behaviors and lack of excessive interference may rather assist her child in the development of autonomy, which in turn psycho-physiologically leads to the stabilization of allergic reactions.

The present study is subject to several limitations. First, the outcome variable disease severity was based on the mother's report of the intensity and frequency of her child's attacks in the past year. Our preliminary investigation indicated that the reports of mothers, most of whom had taken a program to learn about the management of their children's asthma, were in good agreement with the doctors' evaluation, supplemented by information from medical charts, yet they may include some errors originating from the quality of the mother's memory and interpretations. Random errors could have attenuated or even masked the association between the true disease status and the mother's parenting attitudes, stress, and coping styles. An example of a non-random, systemic error would be that a more distressed mother may overestimate her child's asthmatic status or rather become less aware of her child's attacks. This is unlikely, however, because the baseline correlation between the children's disease severity and factors representing the mothers' chronic stress was limited, if any. Second, this study only assessed the parenting styles and stress of mothers, not of fathers. Although all the participating mothers were those who regularly accompanied their children to the hospital and can thus be considered an important care-taker, some fathers may have been even stronger than the mother in affecting the child's disease status. Third, we discussed above the psycho-physiological pathway of the children as a possible linkage between a mother's stress and her child's disease, but this study lacks relevant information such as the children themselves' stress/wellbeing or asthma-associated immunological parameters. Wolf et al reported that parental stress and depression predicted increases in children's inflammatory profiles (eosinophil cationic protein and stimulated interleukin-4 production) over a six month period and that this pattern was not mediated by the child depression and anxiety [32]. Fourth, the limited number of subjects, especially girls, did not allow an analysis stratified by both age category and sex. Future studies with a larger sample of girls should be done for each age cohort to address possible sex differences. Fifth, correlation coefficients between the baseline scores and the scores at follow-up ranged between 0.55 and 0.70 for most of the T-PCRp and SI scales in the present sample, suggesting that the parenting attitudes and stress/coping behaviors that they measure can be dynamic to some degree. Future studies with repeated measures with an interval (e.g., three months) for both psychosocial and outcome variables could address the question how *temporal changes *in a mother's psychosocial factors are associated with their child's disease course.

The present study is among few studies that prospectively tested the hypothesis that certain parenting styles affect the clinical status of asthmatic children or that prospectively addressed the role of parental stress and coping behaviors on the disease status of asthmatic children [8,9], or that showed how a mother's psychosocial properties may affect her child's asthma differently according to the child's age. We addressed *a priori *hypotheses regarding specific parenting styles and parental stress and coping behaviors as risk factors for childhood asthmatic morbidity, used a fairly large sample with a high response rate, used validated instruments for psychosocial factors, and took into account a variety of known or potential confounding factors.

## Conclusions

This study showed that different types of parental stress and coping behaviors may differently affect an asthmatic child's disease status. Specifically, a mother's properties relevant to chronic irritation and anger or emotional suppression predict a worse prognosis for the asthma of younger children, whereas a mother's egocentric behavior predicts a better asthmatic status for older children. As for mothers' parenting styles, too much interference to older children may be harmful to the asthmatic status of the children. Clinicians may benefit by considering an alternative strategy of "treating the parents to heal the child" [13], where the mothers of younger children are advised not to be too worried about if they are falling into "unfavorable" parenting styles, but to pay more attention to the reduction of their own stress. Mothers of older children would be encouraged to increase their own wellbeing via proper egocentric and self-defensive activities, being careful to avoid too much interference with their children.

## Abbreviations

**T-PCRp**: *Ta-ken *Diagnostic Test for Parent-Child Relationship: Parent Form; **SI**: Stress Inventory; **Ig E**: immunoglobulin E; **JPGL**: Japanese Pediatric Guidelines for the Treatment and Management of Asthma; **JSPACI**: Japanese Society of Pediatric Allergy and Clinical Immunology; **OR**: odds ratio; **IQR**: inter-quartile range.

## Competing interests

The authors declare that they have no competing interests.

## Authors' contributions

CKa conceived of the study. JN, CKa and NS designed the study, and CM and HO provided advice on the study design. JN and CKa collected the data, and CM, HO and NS supported the data collection. JN and CKa analyzed the data, and CM provided advice on the data analysis. JN prepared the draft of the manuscript, and CM and NS helped draft the manuscript. SN and CKu supervised the design, data collection and analysis. All authors read and approved the final manuscript.

## Supplementary Material

Additional file 1 **The question about the intensity and frequency of attacks in the past year**. Click here for file

Additional file 2 **Brief description of the T-PCRp scales and the hypothesized associations between a mother's parenting style (T-PCRp scale scores) and the prognosis for her child's asthma**. Click here for file

Additional file 3 **Brief description of the SI scales and the hypothesized associations between a mother's stress/personality factors (SI scale scores) and the prognosis of her child's asthma**. Click here for file

Additional file 4 **Classification of childhood bronchial asthma by severity (JPGL 2000)**. Click here for file
