# Supplementary material for: The parenting attitudes and the stress of mothers predict the asthmatic severity of their children: a prospective study
Source: Biopsychosoc Med. 2010 Oct 7;4:12. doi: 10.1186/1751-0759-4-12 (PMC2959059; doi:10.1186/1751-0759-4-12)
Supplement: Additional file 1 — The question about the intensity and frequency of attacks in the past year. [file 1751-0759-4-12-S1.PDF]

Appendix 1. The question about the intensity and frequency of attacks in the past year

In the past year, what were the intensity and frequency of your child's asthma attacks? Please put up to three circles in the boxes relevant to your child's status. Refer to the guide below the matrix for the intensity of the attacks.

	Intensity of attack		
Frequency of attacks	Major attack	Intermediate attack	Minor attack
Several times or less per year			
Several times per 6 months			
Several times per 1 month			

<u>Guide to evaluating the intensity of the bronchial asthma attacks of children:</u>

Minor attack: wheezy sounds are heard when breathing, though the child looks to be little troubled in playing, talking, eating, and sleeping.

Intermediate attack: child can reply when you talk to them, but he/she looks to be a little troubled in talking, less active and eating less than usual, or his/her sleep may be interrupted by a shortness of breath more than one time during the night; wheezing may be pretty loud.

Major attack: child cannot move for himself/herself, cannot reply when talked to, cannot eat, or cannot lie in bed (*orthopnea*); he/she looks to have much difficulty in breathing and has a poor complexion or poor lip color; wheezing is not necessarily loud.
