# Supplementary material for: The parenting attitudes and the stress of mothers predict the asthmatic severity of their children: a prospective study
Source: Biopsychosoc Med. 2010 Oct 7;4:12. doi: 10.1186/1751-0759-4-12 (PMC2959059; doi:10.1186/1751-0759-4-12)
Supplement: Additional file 2 — Brief description of the T-PCRp scales and the hypothesized associations between a mother's parenting style (T-PCRp scale scores) and the prognosis for her child's asthma. [file 1751-0759-4-12-S2.PDF]

Appendix 2. Brief description of the T-PCRp scales and the hypothesized associations between a mother's parenting style (T-PCRp scale scores) and the prognosis for her child's asthma

The T-PCRp scales*	Brief description or key characteristics	Relation with child's asthma: Hypothesis
Rejective attitudes		
Passive rejection	Neglect; indifference; distrust; ill feeling, inconsistency	●●
Active rejection	Corporal punishment; abuse; threat; over demand; abandonment of care responsibility	●●
Dominating attitude		
Strict control	Strictness; obstinacy; coercion; constant supervision with order, prohibition and criticism	●
Overexpectation	Obtrusion of parent's ambition and demand; disregard of child's disposition, capability and aptitude	●
Overprotecting attitude		
Interference	Overprotection and interference originating from expectations on child	●
Overconcern	Overprotection and interference originating from worry and anxiety about child	●
Obedient attitude		
Sacrifice	Consignment of all power to child; fulfilling child's needs at any cost	●
Doting	Never leaving child at any time; rewarding trivial things; screening child from blame	●●
Inconsistency and disagreement		
Contradiction	Contradictory attitudes to the same behavior of the child	●
Inter-parental inconsistency	Disagreement between parents' attitudes toward the child	●

● and ●●: Predicts poorer prognosis (see text). *The T-PCRp scales belong to one of five typical nurturing attitudes of parent.
