# Supplementary material for: The parenting attitudes and the stress of mothers predict the asthmatic severity of their children: a prospective study
Source: Biopsychosoc Med. 2010 Oct 7;4:12. doi: 10.1186/1751-0759-4-12 (PMC2959059; doi:10.1186/1751-0759-4-12)
Supplement: Additional file 3 — Brief description of the SI scales and the hypothesized associations between a mother's stress/personality factors (SI scale scores) and the prognosis of her child's asthma. [file 1751-0759-4-12-S3.PDF]

Appendix 3. Brief description of the SI scales and the hypothesized associations between a mother's stress/personality factors (SI scale scores) and the prognosis of her child's asthma

The SI scales*	Brief descriptions	Relation with child's asthma: Hypothesis
Group 1: Sense of control over stressful situations		
Low sense of control§	Decreased sense of control over stressful situations leading to hardship, despair, or anger.	●
Group 2: Emotional well-being dependent on other persons and situations		
Object dependence of loss§	Having an important person who causes persistent hopelessness and depression.	●
Object dependence of happiness§	Having a valued person on whom one's happiness is greatly dependent.	
Object dependence of anger	Having a persecuting person who causes chronic irritation and anger.	●●
Annoying barrier	Having a persecuting situation that causes chronic irritation and anger.	●●
Object dependence of ambivalence¶	Repeatedly experiencing ambivalent interpersonal relationships.	●●
Group 3: Telling problems to others and unfulfilled needs for acceptance by others		
Disclosure of negative experiences	A tendency to disclose one's experiences with negative feelings to others.	
Unfulfilled needs for acceptance‡	Chronically having unfulfilled needs for acceptance by others.	●
Group 4: Self-defensiveness in conflicting interpersonal situations		
Altruism‡	An altruistic tendency, accompanied by stress, in interpersonal and social relationships.	●
Egoism¶	A self-defensive, self-interest-oriented attitude in interpersonal and social relationships.	●●
Rationalizing conflicts/frustrations‡	An extreme tendency to rationalize one's interpersonal situations accompanied by conflicts or frustrations.	●
Group 5: Lacking experiences with strong positive and negative emotions		
Lack of emotional experiences	Lack of experiences with strong emotions such as grief, rage, or delight.	

● and ●●: Predicts poorer prognosis (see text). *The SI items and relevant scales were grouped into five in the process of their development.
