# Supplementary material for: The parenting attitudes and the stress of mothers predict the asthmatic severity of their children: a prospective study
Source: Biopsychosoc Med. 2010 Oct 7;4:12. doi: 10.1186/1751-0759-4-12 (PMC2959059; doi:10.1186/1751-0759-4-12)
Supplement: Additional file 4 — Classification of childhood bronchial asthma by severity (JPGL 2000). [file 1751-0759-4-12-S4.PDF]

Appendix 4. Classification of childhood bronchial asthma by severity (JPGL 2000)*

Frequency of attacks	Intensity of attacks		
	Major	Intermediate	Minor
Several times or less per year	Moderate	Mild	Mild
Several times per 6 months	Severe	Moderate	Mild
Several times per 1 month	Severe	Severe	Moderate

Note: The following cases are classified as Severe disease irrespective of the frequency and intensity of fits: (a) cases dependent on systemic corticosteroids administration (excluding inhaled corticosteroids), (b) cases who experienced a major attack with consciousness disturbance. *Developed by the Japanese Society of Pediatric Allergy and Clinical Immunology.
